# The art of building bone: emerging role of chondrocyte-to-osteoblast transdifferentiation in endochondral ossification

**DOI:** 10.1038/s41413-018-0021-z

**Published:** 2018-06-14

**Authors:** Patrick Aghajanian, Subburaman Mohan

**Affiliations:** 1 Musculoskeletal Disease Center, Veterans Affairs Loma Linda Healthcare System, Loma Linda, California USA; 2 Department of Medicine, Loma Linda University, Loma Linda, California USA; 3 Department of Orthopedics, Loma Linda University, Loma Linda, California USA; 4 Department of Biochemistry, Loma Linda University, Loma Linda, California USA

## Abstract

There is a worldwide epidemic of skeletal diseases causing not only a public health issue but also accounting for a sizable portion of healthcare expenditures. The vertebrate skeleton is known to be formed by mesenchymal cells condensing into tissue elements (patterning phase) followed by their differentiation into cartilage (chondrocytes) or bone (osteoblasts) cells within the condensations. During the growth and remodeling phase, bone is formed directly via intramembranous ossification or through a cartilage to bone conversion via endochondral ossification routes. The canonical pathway of the endochondral bone formation process involves apoptosis of hypertrophic chondrocytes followed by vascular invasion that brings in osteoclast precursors to remove cartilage and osteoblast precursors to form bone. However, there is now an emerging role for chondrocyte-to-osteoblast transdifferentiation in the endochondral ossification process. Although the concept of “transdifferentiation” per se is not recent, new data using a variety of techniques to follow the fate of chondrocytes in different bones during embryonic and post-natal growth as well as during fracture repair in adults have identified three different models for chondrocyte-to-osteoblast transdifferentiation (direct transdifferentiation, dedifferentiation to redifferentiation, and chondrocyte to osteogenic precursor). This review focuses on the emerging models of chondrocyte-to-osteoblast transdifferentiation and their implications for the treatment of skeletal diseases as well as the possible signaling pathways that contribute to chondrocyte-to-osteoblast transdifferentiation processes.

## Cell biology: When cartilage becomes bone

A basic principal of cell differentiation is that cells become increasingly specialized until they reach a fixed terminal state - yet recent studies suggest that mature cells can, and do, change into new types. Such ‘transdifferentiation’ seems to occur in many different tissues, but in this review, Patrick Aghajanian and Subburaman Mohan at VA Lorna Linda Healthcare System focus on the transition of cartilage to bone. Although a similar transition is well-known to occur during fetal development and fracture healing, in both cases, mesenchymal stem cells brought in by invading blood vessels were thought to differentiate into bone-producing cells. However, recent studies suggest that cartilage cells can themselves transdifferentiate into bone cells. A better understanding of this process could lead to new therapies to boost fracture healing and tackle bone-wasting disorders.

## Introduction

The process of cell differentiation is a widely studied phenomenon which is the basis for all developmental processes. The basic underlying principle of how cell differentiation proceeds is that with each step of the differentiation pathway, cells become programmed to follow a certain specified lineage progression until they are terminally differentiated with an end point of apoptosis or cell death.^1–3^ Recently, however, many studies have introduced the idea of transdifferentiation, the differentiation of cells (terminally differentiated or not) to a cell type that does not follow the normal, preprogrammed differentiation mechanism. Transdifferentiation refers to a process where one mature cell switches its phenotype and function to that of another mature differentiated cell type.^4–8^ This process occurs via two main mechanisms. The first is via direct transdifferentiation of one tissue type to another without undergoing an intermediate pluripotent state or becoming a progenitor cell, which will be denoted as direct transdifferentiation. The second major method occurs via an intermediate step, often manifested by a dedifferentiation and redifferentiation. This mechanism will be denoted as intermediate transdifferentiation.

Cell transdifferentiation has been described in the literature in multiple tissue types and model organisms, and is therefore neither a species- nor tissue-specific phenomenon. There are now many examples of this phenomenon, but for this review, we will only use a small representative population. In the *Drosophila* intestine, Takashima et al. have shown that ectodermally derived hindgut cells migrate anteriorly to the midgut to form epithelial cells of the endodermally derived midgut, becoming indistinguishable from the surrounding epithelial cells.^9^ In zebrafish, during normal myocardial regeneration, Zhang et al. observed that a population of atrial cardiomyocytes can migrate to the ventricle and repair it by direct atrial-to-ventricular transdifferentiation and that this mechanism is regulated by notch signaling.^10^ Not surprisingly, tissue regeneration in amphibians and reptiles can also use transdifferentiation mechanisms. *Xenopus* eye lenses, when removed, can regenerate as the result of transdifferentiation of corneal epithelium to lens cells.^11^ Further studies have shown that upregulation of BMP and WNT^12^ as well as matrix metalloproteases^13^ are required for this direct transdifferentiation.

Furthermore, there are examples of chondrogenic tissues transdifferentiating into cell types of different origins and vice versa. Atherosclerotic lesions in mice have presented what seems to be the transdifferentiation of vascular smooth muscle to chondrogenic tissue. This was confirmed by the reduced expression of α-smooth muscle actin and the increased expression of SOX-9, a marker for immature chondrocytes, in the calcified lesions,^14^ as well as a different study that documented this event to occur via increased expression of tissue non-specific alkaline phosphatase and BMP-2 activation.^15^ In a reversal of roles, rat chondrocytes have been shown, when stimulated by neurogenic growth factors (FGF-2, Neurobasal-A, EGF, and IGF-1), to transdifferentiate into stellate neuronal cells with ablation of COL2 expression and expression of neuron-specific proteins such as NF-200, MAP-2, and β-III tubulin.^16^ Another case of osteogenic transdifferentiation involves the dedifferentiation of myoblasts via BMP-2 induction of SMAD1, which is mediated by osteoactivin. Osteoactivin, in turn, downregulates myogenic markers and upregulates osteogenic markers, such as RUNX2 and ALP.^8,17–20^ Finally, human gingivial fibroblasts have been shown, both in vitro and in vivo to transdifferentiate to osteoblastic lineage cells when treated with 5-aza-2′-deoxycytidine followed by subsequent treatment with BMP2. This was confirmed in vitro by upregulation of *Runx2* and *Alp* expression and in vivo subcutaneous transplantation into mouse, which resulted in increased bone mineral content and bone volume/tissue volume.^21^ The issue of whether the transdifferentiation observed in some of these in vitro studies can be attributed to the resident mesenchymal stem cells present in the cultures used remains to be examined.

Beyond these examples, it is important to note that any process of developing induced pluripotent stem cells from somatic cells is a form of intermediate transdifferentiation. For example, in the famous study by Takahashi and Yamanaka, mouse embryonic fibroblasts were transduced to express what are now known as the Yamanaka factors (*Oct3/4*, *Sox2*, *c-Myc*, *Klf4*) to induce a pluripotent cell intermediate. These cells were then able to form teratomas in cell culture settings and when introduced into an undifferentiated blastocyst, were able to follow normal differentiation programming indistinguishable from the natural pluripotent stem cells.^22^ Transdifferentiation is, therefore, not limited to artificial cell culture settings, but a natural phenomenon.

While there are many examples of transdifferentiation in the literature, this review will focus mainly on recent examples of transdifferentiation relating to the transformation of chondrogenic tissue into osteogenic tissue and the underlying mechanisms.

## Vascular source of osteogenic precursors in the canonical pathway of endochondral ossification

**Fig. 1:**
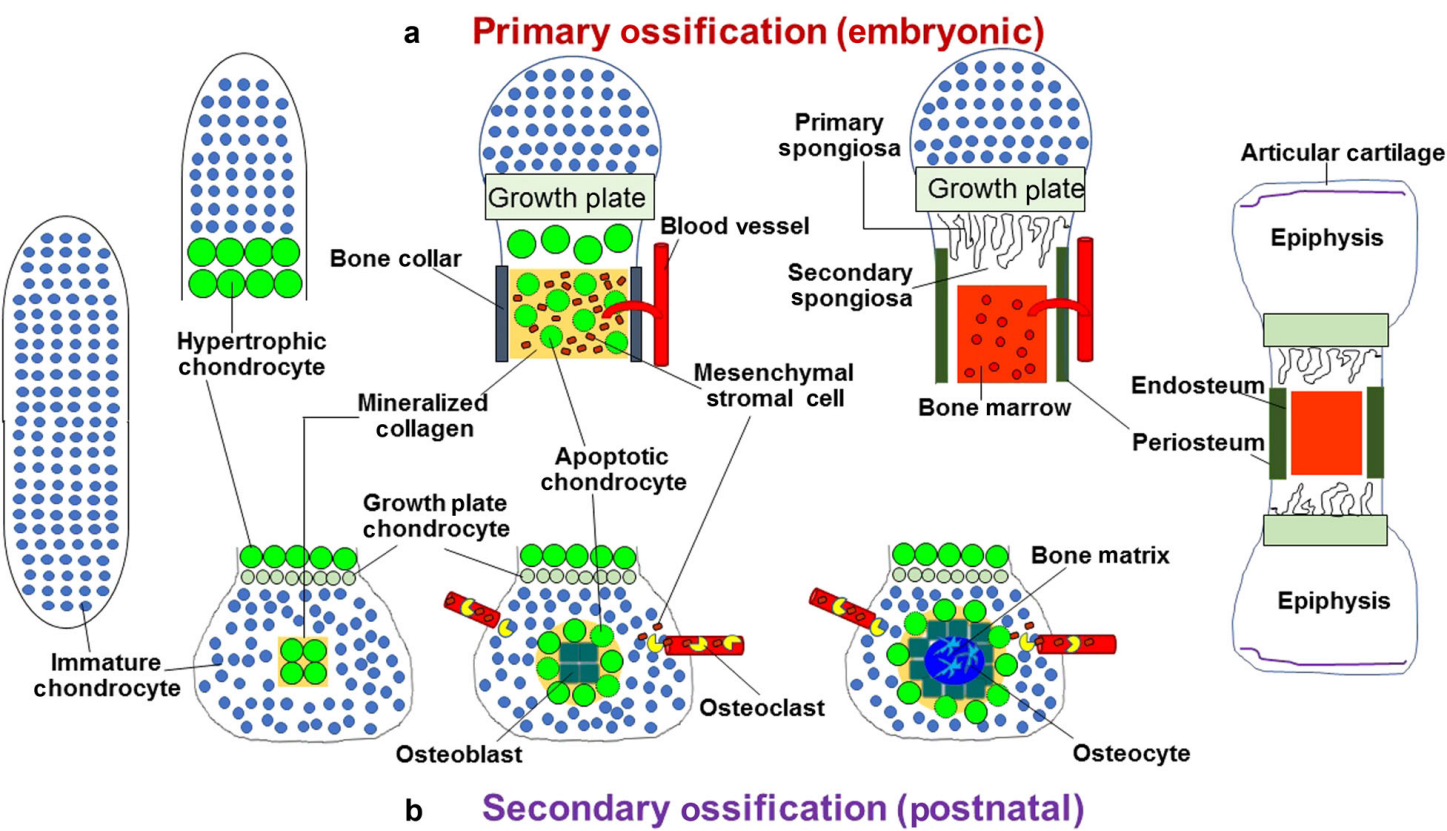
Existing paradigm for long bone endochondral ossification. **a** Primary endochondral ossification begins with the formation of a chondrocyte template during embryogenesis. Chondrocytes undergo hypertrophy beginning from the mid-diaphysis, eventually extending to the epiphyseal poles. Vasculature invades the forming bone, transporting marrow, mesenchymal stromal cells, and osteoclasts. Hypertrophic cells undergo apoptosis, aided by the removal of matrix by osteoclasts. Mesenchymal stromal cells differentiate into osteoblasts and then osteocytes. **b** Secondary ossification occurs at the epiphysis post-natally in rodents. Immature chondrocytes at the center of the epiphysis become hypertrophic to produce mineralized collagen and eventually undergo apoptosis. Vasculature invades and transports marrow, mesenchymal stromal cells, and osteoclast precursors. Bone formation initiates at the center and extends peripherally.

**Fig. 2:**
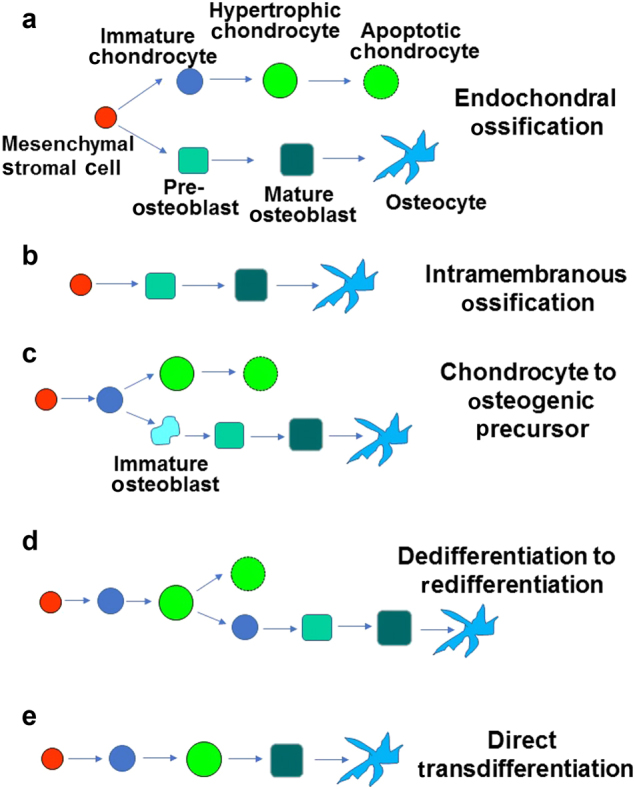
Models of bone formation. **a** Endochondral ossification. Mesenchymal stromal cells develop into two different lineages, chondrogenic and osteogenic with no other intermediates. **b** Intramembranous ossification. Osteoblast development does not require the formation of a chondrocyte template. Mesenchymal stromal cells directly differentiate in an osteogenic lineage. **c** Chondrocyte to osteogenic precursor. Immature chondrocytes differentiate into an osteogenic precursor population which then differentiate into pre-osteoblasts and osteoblasts. **d** Dedifferentiation to redifferentiation. Hypertrophic chondrocytes dedifferentiate into immature chondrocytes, which directly differentiate to an osteogenic fate. **e** Direct transdifferentiation. Hypertrophic chondrocytes directly differentiate to osteoblasts.

To understand the different mechanisms of chondrocyte-to-osteoblast transdifferentiation, it is first important to reference the traditional canonical mechanism of endochondral ossification.^23–28^ Endochondral ossification, in the long bones, is the process of replacing cartilage with bone (Figs. 1 and 2a). This process differs in various bone tissues. In the long bones themselves, there are different temporo-regional mechanisms of endochondral ossification. These processes are referred to as primary endochondral ossification (Fig. 1a) and secondary ossification (Fig. 1b). Primary ossification begins at embryonic day (E) 14.5–15.5 in rodents and encompasses the formation of the periosteum, the bone collar (osteoid layer), and trabeculae which begins at the mid-diaphysis and extends to the growth plates. Mechanistically, the most accepted mechanism for these models begins with the formation of a template of rapidly proliferating immature chondrocytes, which secrete a type 2 collagen (COL2) matrix and make up both the perichondrium and the immature chondrocytes of the diaphysis. This matrix is then degraded when chondrocytes undergo hypertrophic differentiation and secrete enzymes such as matrix metalloprotease 13 (MMP13) and ADAM-TS4.^27,29–31^ Chondrocytes then begin to proceed through apoptosis and the perichondrium becomes periosteum, while the inner layer of the periosteum undergoes intramembranous ossification to form bone collar which encloses the chondrocytes in the center of the bone. Concurrently, the tissue is invaded by incoming vasculature. Osteoclasts from the invading vasculature remove chondrocytes from the template and mesenchymal stromal cells replace apoptosing chondrocytes.^27,32^ Mesenchymal stromal cells then differentiate into osteoblasts to produce type 1 collagen (COL1) and other bone matrix proteins such as bone sialo protein (BSP) and osteocalcin which stimulate bone mineralization.^33,34^ Secondary endochondral ossification occurs in a very similar manner. The principal differences between primary and secondary ossification are the time at which they occur. Secondary ossification initiates at post-natal day (P) 7–8 in rodents in the mid-epiphysis, and then expands peripherally to widen the bone, while primary ossification is involved more in longitudinal growth.^26,35^ Furthermore, secondary ossification largely lacks a periosteal layer and ossification of both ends of the long bone epiphysis may occur at different time points, as the proximal femur epiphysis develops bone much later than the distal femur epiphysis in humans.

As mentioned earlier, endochondral ossification is not the only method by which bone formation occurs. Intramembranous ossification plays an important role in the formation of flat bones in the skull, mandible, and clavicle.^36,37^ More importantly, as it relates to the long bones, intramembranous ossification is responsible for the formation of the subperiosteal surface. Instead of first forming a cartilage intermediate which is then replaced by bone via a tightly coupled process involving chondrocyte, osteoblast, and vascular differentiation, periosteum is formed directly by differentiating mesenchymal cells. These cells then differentiate directly into osteoblasts and secrete COL1 and proteoglycans to form an osteoid matrix, which is then mineralized to form the bone^38^ (Fig. 2b). Mesenchymal stromal cells which are enclosed by the osteoid layer differentiate into osteocytes, including cells of the periosteum. Bone formed via endochondral ossification does not form nodules, and requires a chondrogenic template. Furthermore, intramembranous ossification does not require chondroclasts or osteoclasts for initial remodeling.

Endochondral ossification is also involved in the natural healing of bone fractures. Although fracture healing involves both intramembranous and endochondral ossification, the formation of cartilaginous callus, which later undergoes mineralization and resorption for subsequent replacement with bone, represents the primary method by which fracture healing proceeds. After fracture, the blood clots form a hematoma, which is then stabilized by the surrounding periosteum and other tissues. The inner layer of the periosteum, or inner cambium, produces a mass of chondrocytes to form a template very similar to developmental endochondral ossification. These cells then proceed through normal ossification and form both a hard and soft callus in place of the fracture.^39,40^ It is traditionally accepted that the primary source of osteoblasts during endochondral ossification both during skeletal development and fracture repair is through invading blood vessels that bring in mesenchymal stem cells which proliferate and differentiate to become osteoblasts. However, in contrast to the canonical pathway involving chondrocyte apoptosis and perivascular location as the origin of mesenchymal stem cells, we and others have recently found that chondrocytes undergo transdifferentiation into bone-matrix-producing osteoblasts both during normal endochondral ossification as well as during fracture repair.

The classic model of endochondral ossification emphasizes the apoptotic fate of terminally differentiated chondrocytes which was first suggested by characteristic changes in morphology, and, more recently, by the pattern of DNA fragmentation and other characteristic features of apoptosis.^17,41–43^ Based on the findings from several laboratories that the rate of chondrocyte apoptosis increased during fracture healing, it is generally accepted that hypertrophic chondrocytes are programmed to die during the process of endochondral ossification. However, other studies have indicated evidence for transformation of hypertrophic chondrocytes into osteoblasts or other cell types. In this regard, our previous study revealed that Bax deficiency resulted in increased cartilage production that is caused by increased proliferation but without changes in apoptosis.^44^ Furthermore, we and others^44–46^ have found that the change in apoptotic rate of chondrocytes in response to fracture is rather small (approximately 5%), thus not excluding other fates of hypertrophic chondrocytes such as transdifferentiation into osteoblasts.

## Chondrocyte-to-osteoblast transdifferentiation models

As mentioned earlier in this review, the canonical bone development model involves the differentiation of mesenchymal stem cells into a specific cell lineage fate. Typically, depending on the time and signaling, a mesenchymal stromal cell will differentiate through one specific lineage, either chondrogenic, adipogenic, or osteogenic. The end of each will result in terminal differentiation (Fig. 2a). In this model, chondrocytes that hypertrophy will typically eventually apoptose.^27,32^ Beyond this, there are currently three known major models of transdifferentiation of chondrocytes to osteoblasts, and each are mechanistically different and may be specific for different skeletal sites during embryonic and post-natal growth periods. We will differentiate each model by designating them with the method by which the chondrocytes differentiate. The first two models of which imply intermediate transdifferentiation and the final model which implies direct transdifferentiation.

**Fig. 3:**
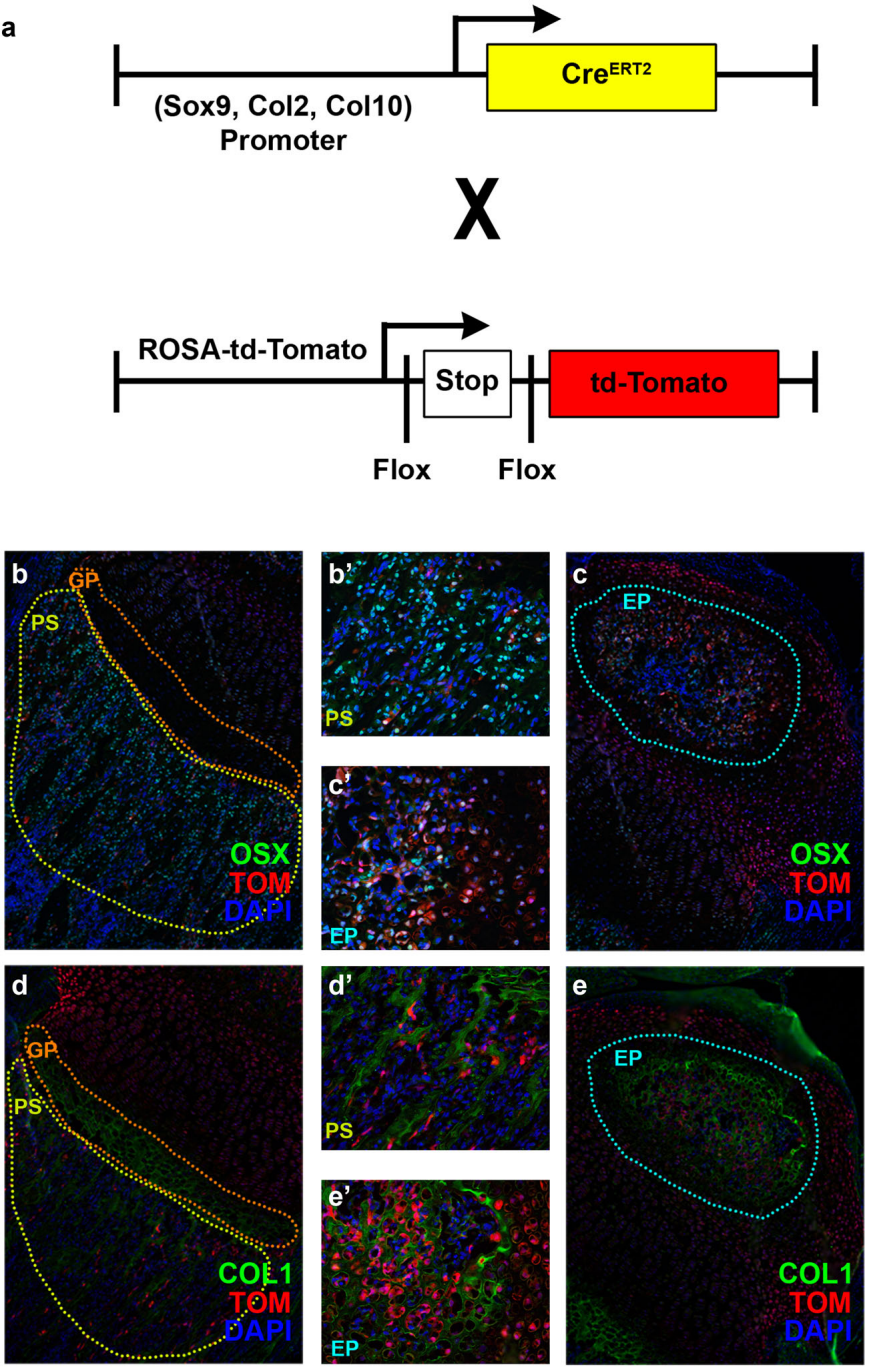
Lineage tracing of chondrocytes to osteoblasts. **a** The lineage trace construct consists of a tamoxifen-inducible CRE, which is driven by a promoter of a chondrogenic tissue, such as *Sox9*, *Col2*, *Col10*, or *Acan*. This in turn excises the stop codon blocking the *ROSA-LSL-TdTomato* construct. All further cells in the recombined lineage will express td-tomato. **b**, **b**′ *ROSA-LSL-TdTomato* floxed mice, positive for *Col2-Cre*^*ERT2*^, were administered with tamoxifen at P3 and mice euthanized at P10. Coexpression of OSX and Tomato-red in cells of the primary spongiosa. **c**, **c**′ Coexpression of OSX and Tomato-red in cells of the epiphysis. **d**, **d**′ Coexpression of COL1 and Tomato-red in cells of the primary spongiosa. **e**, **e**′ Coexpression of COL1 and Tomato-red in cells of the epiphysis. COL1, collagen type 1; OSX, osterix; TOM, Tomato-red; DAPI, nuclear stain; PS, primary spongiosa; GP, growth plate; EP, epiphysis.

The chondrocyte to osteogenic precursor (OP) model suggests that immature chondrocytes in the growth plate can differentiate into a transient osteogenic precursor in the metaphysis.^47^ During rapid bone growth, this provides a mechanism for generation of both stromal cells and osteoblasts (Fig. 2c). In this model, the transient osteogenic precursors are not thought to self-renew. In addition, this model seems to be specific for metaphyseal growth, much of which occurs embryonically during primary ossification, but can extend into post-natal growth and are separate from adult mesenchymal progenitors. Experimental support for this idea is drawn from fate mapping studies in which cells were lineage traced using a tamoxifen-inducible CRE preceded by promoters for genes expressed by immature chondrocytes such as *Col2*, aggrecan (*Acan*), and SRY-Box9 (*Sox-9*) to induce expression of the red fluorescent protein variant TdTomato (Fig. 3a). Pulse chase tamoxifen experiments during embryonic development suggested that cells originating from *Osx-CreER* proliferate and persist for a brief time in the primary spongiosa and metaphysis, only to disappear, while those originating from *Col2-CreER* animals continually form bone in the perichondrium, primary spongiosa, and secondary ossification centers, supporting the idea of a transient intermediate. In this study, the authors speculate that the early mesenchymal progenitors expressing chondrocyte markers provide a rapid source of osteoblasts during the rapid phase of bone growth, but are different from adult mesenchymal progenitors.^48^

The dedifferentiation to redifferentiation model provides an alternate view of chondro-osteogenic transdifferentiation. In this model, chondrocytes hypertrophy and either enter apoptosis or dedifferentiate first into immature chondrocytes, and then redifferentiate into osteoblasts and further into osteocytes (Fig. 2d). This model has been described to occur during embryonic and post-natal development, but also during fracture healing.^49^ Specifically, Zhou et al. implied in their experiments that chondrocytes transdifferentiate into osteoblasts and contribute to longitudinal growth in long bones. In this work, the authors used a tamoxifen-inducible *Acan-CreERT2* or *Col10-Cre* (a marker typically used to identify hypertrophic chondrocytes) to drive the expression of a *ROSA-LSL-TdTomato* marker, this was coupled with a *2.3Col1-GFP* reporter to show that, in fact, the osteoblasts lineage arose from the transdifferentiating hypertrophic chondrocytes. Furthermore, *Acan-CreERT2; ROSA-LSL-TdTomato* cells expressed *2.3Col1-GFP* at the ossified fracture calluses at 14 days post-surgery, suggesting that a chondrocyte-to-osteoblast transdifferentiation mechanism is at least somewhat involved in fracture healing. This mechanism was confirmed by another group using both *Acan-CreERT2* and *Col2-CreERT2*.^50^ Additionally, Hu et al. reported the expression of stem-like markers (OCT4, NANOG, SOX2) in chondrocytes located in the fracture callus, suggesting the possibility of dedifferentiation.^50^ In a subsequent study, the model of dedifferentiating chondrocytes was further proposed using a tamoxifen-inducible *Col10-ERT2* to drive YFP expression, and then colabeled with markers typically found in osteogenic cells (COL1, OSX) during mandibular growth, further solidifying evidence that osteoblasts are able to arise from hypertrophic chondrocyte lineages.^51^ In another study, Yang et al.^52^ demonstrated using *Col10-CreERT2* that hypertrophic chondrocytes may become *Col1a1*-expressing osteoblasts and sclerostin-expressing osteocytes in prenatal and post-natal bones and during bone injury repair. While these studies certainly suggest the possibility of hypertrophic chondrocytes dedifferentiating first before differentiating into osteogenic tissue, this hypothesis has been left open for interpretation.

Differentiation of stem cells into specialized cells requires an upregulation of genes involved in the creation of a specific cell phenotype and suppression of genes responsible for cell stemness.^53^ In a recent study, Kang et al.^54^ evaluated the expression of 11 stemness genes during in vitro differentiation of induced pluripotent stem cells into mesenchymal stem cells and tri-lineage (osteoblast, chondrocyte, and adipocyte) differentiated cells. They found that while all stemness genes were expressed in induced pluripotent stem cells, most of the stemness genes except Klf4 and C-Myc were not expressed in the tri-lineage differentiated cells. These findings are in favor of use of stemness gene expression marker to identify osteogenic stem cells. However, the issue of whether the identified stem cell marker gene expression is in dedifferentiated hypertrophic chondrocytes per se and not in the contaminating stem cells present in tissue preparations examined needs to be carefully investigated before concluding the involvement of an intermediate dedifferentiation step during osteogenic differentiation of hypertrophic chondrocytes.

Finally, the direct transdifferentiation model suggests that direct transdifferentiation is the method by which post-natal secondary ossification occurs. The direct transdifferentiation model suggests that chondrocytes will mature and hypertrophy, and that they do not apoptose, but instead differentiate directly into osteoblasts and subsequently into osteocytes (Fig. 2e). In a recently published study, we administered tamoxifen at post-natal (P) day 3 to *Col2-CreERT2* animals to activate the *tdTomato* reporter in early immature chondrocytes. These cells then proceed to present themselves in newly formed bone of the epiphysis by co-localizing with bone markers such as OSX, BSPII, ALP, DMP1, OCN, and COL1. Prior to this, these same cells will express markers typically seen in hypertrophic chondrocytes such as COL10 and MMP13. The *tdTomato* marked cells have been found to be embedded in bone matrix as osteoblasts and osteocytes after several days (Fig. 3b-e**′**) or weeks^4^ of post-natal development. This process all initiates before vascular invasion of the epiphysis reaches the region of secondary ossification. Furthermore, there is no observed increase in proliferation or apoptosis observed in the epiphyseal hypertrophic chondrocytes. Coupled with the co-localization data with bone markers, transdifferentiation seems to be an important mechanism for early post-natal bone formation. Even a study as early as 1992 in chick embryos suggested that hypertrophic chondrocytes have the capability to transdifferentiate into bone-matrix-forming cells.^55^ Consistent with this data, we demonstrated that treatment of ATDC5 chondrocytes with thyroid hormone increased the expression levels of osteoblast differentiation markers and bone nodule formation in vitro, thus providing evidence for chondro-osteoblast transdifferentiation.^56^ Based on these in vitro findings and our in vivo findings that hypertrophic chondrocytes express markers of osteoblasts, our model of direct transdifferentiation also suggests that hypertrophic chondrocytes may, in fact, be a misnomer, and that they are in fact pre-osteoblasts. This model is also supported by experiments using a *Col10-CreERT2* to drive fluorescent marker lineage trace of hypertrophic cells suggested that not only did the cells of the growth plate contribute to the primary spongiosa, but that these cells also seemed to directly transdifferentiate.^57^ A possible indicator of this is embodied by the expression of OSX, a marker for early osteoblasts that often manifests in late stage hypertrophic chondrocytes. Moreover, this model suggests that direct transdifferentiation is the most likely mechanism for the formation of epiphyseal osteoblasts as the process of dedifferentiation and redifferentiation would not occur quickly enough in the span of time in which the bone appears immediately after the formation of the hypertrophic chondrocytes in the epiphysis.^4,56^ Direct transdifferentiation is not unique to secondary ossification as it probably also occurs during primary ossification as well as during fracture healing.

In a recent study, Sakagami et al.^58^ have evaluated if *Col2α1*-expressing cells contributed various models of ossification occurring during the craniofacial skeletal complex by an in vivo cell mapping technique utilizing *Col1α1(2.3* *kb)-GFP* and *Col2α1-Cre:ROSA-LSL-tdTomato* mice. They found that *Col2α1-Cre*, as expected, consistently marked most skeletal cells in the bones of cranial base, which primarily form by endochondral ossification route. However, virtually all *Colα1-GFP*^+^ osteoblasts near the suture were green, suggesting that they were not derived from *Col2α1-Cre*-marked cells. In contrast, many osteoblasts in the inner aspect of the calvaria were marked with *Col2α1-Cre*, thus suggesting that mechanisms of craniofacial bone formation may be complex and utilize both *Col2α1* positive and negative early progenitors of the skeletal lineage. Thus, while these and other studies raise the possibility that the early progenitors expressing *Col2α1* represent common precursors and could become osteoblasts or chondrocytes, in the context of this review, transdifferentiation refers to the conversion of fully differentiated hypertrophic chondrocytes into osteoblasts rather than undergoing their preprogrammed cell death.

## Signals that control chondrocyte-to-osteoblast transdifferentiation

In order to properly investigate the process of transdifferentiation, it is important to define the signals involved in specifying the phenotype of varying cell types to discern how much of each of the signaling processes are conserved in different tissues. While signaling may play a role in causing cell differentiation, at times, the lack of a signal also plays a role. In the example of hindgut to midgut transition in *Drosophila*, the adult hindgut progenitors rely on wingless (WG) signaling (a homolog of WNT) to keep them in an undifferentiated state. Once cells of the anterior hindgut proliferation zone (HPZ) migrate anteriorly, they eventually cross a threshold that prevents cell renewal, and instead are exposed to other molecular signals that induce this transition. Moreover, cells of the HPZ express *GATAe* which is required to induce migration and subsequent loss of hindgut fate markers. Additionally, in *Wg*-overexpressing animals, this event does not occur, and in fact, the cells do not even migrate.^9^ *Wnt* also plays a role in the induction of other cell types into the osteogenic lineage, as well as, inhibiting osteogenic cells from transdifferentiating into chondrogenic or adipogenic lineages.^59–65^ This is much different than the specific signals noted here for chondrocyte-to-osteoblast transdifferentiation, but other examples, such as the formation of iPSCs, require factors like *Oct4* and *Sox2* expression, which was also one of the factors involved in the dedifferentiation described in a paper using the DR model of chondrocyte transdifferentiation.^22,50^

While it is not known exactly what causes the transdifferentiation of chondrocytes into osteoblasts, it seems that the expression of some factors is necessary for that transition. Factors such as IGF-1 are known to be important in regulating chondrocyte and osteoblasts proliferation and maintenance.^66–68^ IGF-1 also regulates osteoblast differentiation from mesenchymal stromal cells through mTOR.^69^ Knockouts of IGF-1 also reduce the anabolic effects of important osteogenic hormones such as parathyroid hormone (PTH).^70,71^ Perhaps it has a role to play in chondrocyte-to-osteoblast transdifferentiation, but that remains to be seen. Teriparatide, a recombinant of PTH approved for clinical use, has been shown to increase proliferation and differentiation of *Sox9-cre* chondrogenic precursors to osteogenic fates, that it functions through the PTH receptor, and that upon withdrawal of teriparatide treatment, these precursors do not undergo chondrogenic differentiation, but instead develop into an adipogenic lineage.^72^ The importance of WNT signaling in endochondral ossification is well established by studies on disruption of *β-catenin* specifically in chondrocytes.^60,63,73^ In terms of mechanisms for regulation of IGF-I and WNT signaling during endochondral ossification, it is known that thyroid hormone levels begin to spike at late pre-natal and early post-natal growth, which are key bone-forming time points.^56,74^ This rise in thyroid hormone levels during the second week of post-natal life is indispensable for the IGF-I and *β-catenin* expression in the epiphysis.^4,56^ Further, the expression of *Runx2* and its downstream effector *Osx* are essential for ossification to occur in the epiphysis and trabecular bone.^75^ *Osx* is also an important regulator in cementogenesis,^76^ a process that is very similar to that of bone formation.^77,78^ In the case of cementogenesis, *Osx* is a major downstream regulator of the *Wnt*/*β-catenin* signal transduction pathway and is absolutely necessary for this process to proceed.^76,79^ Absence of circulating thyroid hormone which functions through modulation of *Osx* and Indian hedgehog (*Ihh*) expression has been observed to prevent bone formation in the epiphysis. In our recent work, we have shown that thyroid hormone-deficient animals could form prehypertrophic chondrocytes, but bone formation was halted during normal ossification windows, and in later stages was dysfunctional. Much of this was related to the inability of chondrocytes to transdifferentiate into osteoblasts.^56^ An important role for calcium and its putative receptor, calcium sensing receptor, in transdifferentiation can also be speculated based on the established effects of extracellular calcium in promoting the differentiation of chondrocytes and osteoblasts.^80–82^

In an example of lens regeneration in newts using cells from other tissues, the transdifferentiation of the epithelial cells of the dorsal iris into lens cells was found to increase expression of both Sonic hedgehog (*Shh*) and *Ihh*.^83^ IHH has been shown to be an important factor for differentiation of immature chondrocytes into a hypertrophic state by regulating levels of parathyroid hormone-related protein (PTHrP), which keeps chondrocytes in an immature proliferative state.^64,84^ IHH also activates GLI2, which activates osteogenic differentiation.^85–92^ This is mediated in part through the GLI2 activation of *Runx2* and *Osx* expression.^56,91^ Once the correct balance of IHH expression is achieved, chondrocytes and mesenchymal cells can differentiate. As suggested in Aghajanian et al., IHH-mediated *Gli2* expression is a very likely mediator of osteogenic transdifferentiation of chondrocytes.^4^ This point is further corroborated in a study that suggests IHH and GLI2 upregulate *Runx2*, *Alp*, and *Ocn* expression along with increased bone mineralization. Moreover, this effect was not observed by *Gli3* overexpression and *Gli2* dominant negative animals inhibited the IHH dependent effect on bone formation.^91^ Investigators in another study observed the coexpression of both bone (*Osx*, *ColI*, *Runx2*) and cartilage (*Sox9*, *Col2*, *Col10*) genes during jawbone repair in zebrafish, suggesting a transdifferentiation program. Moreover, in *Ihh*^−*/*−^ zebrafish, callus formation after fracture is greatly reduced, as is bone mineralization and periosteal bone formation, while chondrocyte proliferation in the fracture region remains unaffected.^93^ Finally, *Ihh* expression has been shown to increase under the influence of thyroid hormone expression (a known inducer of bone formation), and specifically through thyroid hormone receptor β1 (TRβ1). Direct interaction between *Ihh* and TRβ1 was confirmed via the presence of a thyroid hormone response element (TRE) in the promoter of the *Ihh* gene.^94^

**Fig. 4:**
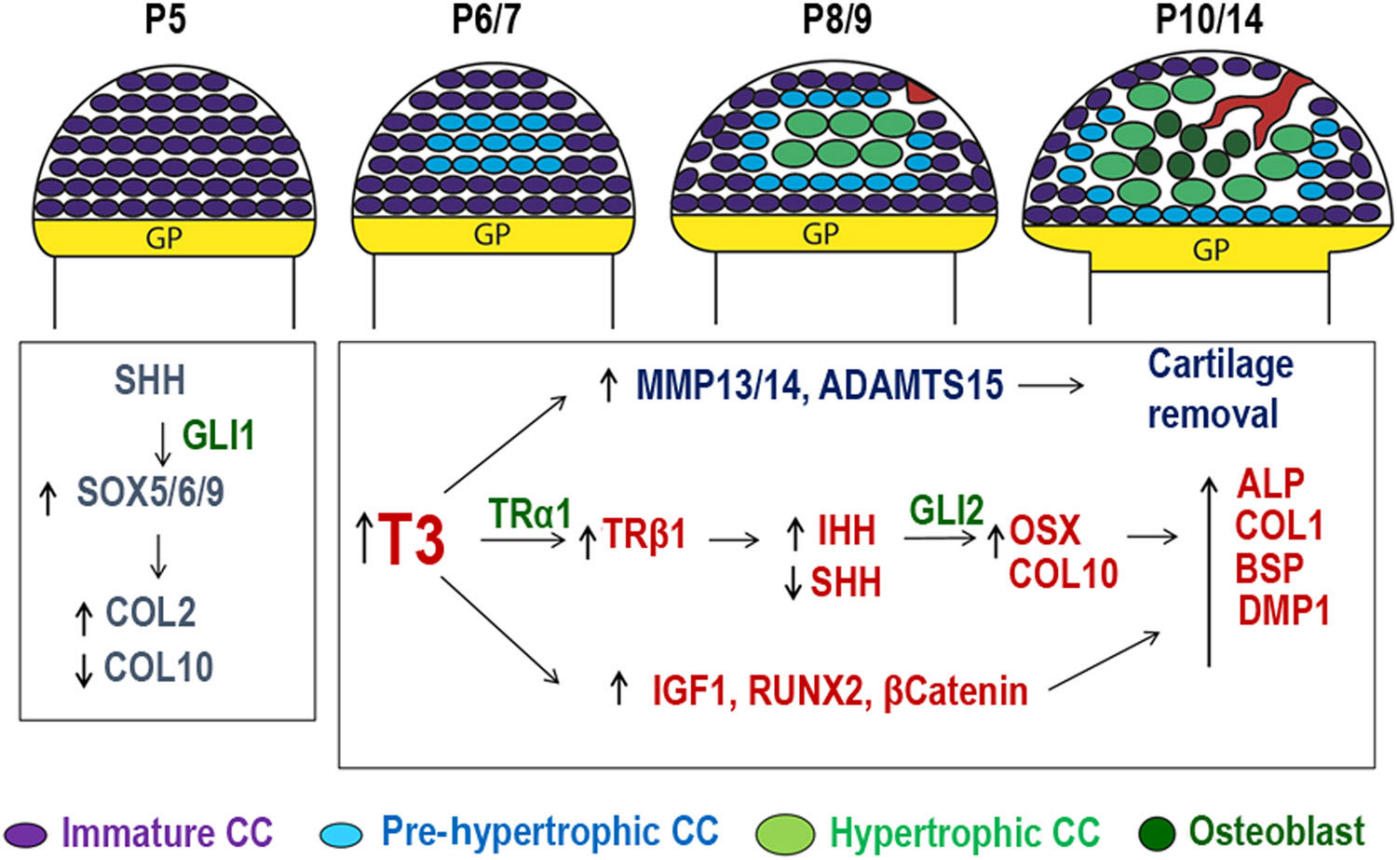
Proposed model for thyroid hormone-mediated early post-natal development of the secondary ossification center (SOC). During embryonic and early post-natal development when thyroid hormone (TH) levels are low, epiphyseal chondrocytes express elevated levels of SHH, which acts through GLI1 to maintain these cells in proliferative immature state by activating Sox5/6/9 transcriptional activity. At P6/P7, rise in TH increases TRβ1expression and thereby IHH expression. IHH acts through GLI2 to decrease SOX9 and COL2 expression, while MMP13 and ADAMTS5 expression increases to deplete the COL2 matrix. Pre-hypertrophic chondrocyte (CC)s begin to express COL10 and OSX in the P8/P9 period. These in turn activate DMP1 and ALP in the SOC, meanwhile blood vessels invade from the periphery of the articular CCs. Text and image obtained from ref. ^4^

*Runx2*, a known factor in osteogenic differentiation, was thought to be important in the transdifferentiation process in the chondrocyte to osteogenic precursor model.^47^ The role of *Runx2* as a master regulator of osteogenic fate has been further implicated in the process by its important role in the transdifferentiation of adipocytes,^95^ primary skeletal myoblasts,^96^ odontoblasts,^97^ and vascular smooth muscle cells^59^ to osteogenic cell types. *Runx2* activation usually comes in conjunction with the usual secreted factors such as bone morphogenic proteins (BMPs) or WNTs. An example of this is the transdifferentiation of myoblasts into osteoblasts via induction by both RUNX2 and BMPs. Naturally, BMPs have the ability to not only upregulate the expression of osteogenic factors, but also downregulate expression of myogenic factors.^18^ As it pertains to transdifferentiation, Cho et al. indicated that BMP2 is an important regulator of osteogenic transdifferentiation from gingivial fibroblasts.^21^ In our studies, we found that thyroid hormone upregulated expression levels of many growth factors (IHH, BMP, IGF-I, and RANKL) as well as transcription factors (RUNX2, OSX, β-CATENIN) during epiphyseal bone formation, thus suggesting involvement of multiple signaling pathways in driving the chondrocyte–osteoblast transdifferentiation process (Fig. 4). Additional in vivo studies using appropriate animal models are needed to determine the relative contribution of the various signaling pathways identified in in vitro studies in regulating chondrocyte-to-osteoblast transdifferentiation.

### Implications for clinical practice

The clinical relevance of understanding the potential contribution of the chondrocyte-to-osteoblast differentiation route to endochondral ossification and the molecular mechanisms that contribute to the switch from chondroblastic to osteoblastic cellular machinery are as follows. 1) The current therapies for non-union fractures involve strategies that utilize mesenchymal stem cells-derived from patients and/or growth factors to promote direct bone formation at the fracture site. However, these strategies have not been proven to be effective and may be cost prohibitive. Since chondrocyte proliferation and differentiation predominate early during the fracture repair process, it may be more prudent to expand the existing chondrocytes and convert them to osteoblasts capable of performing all of the functions required to resorb cartilage and produce bone as performed by the epiphyseal chondrocytes in secondary ossification centers when thyroid hormone levels are high. Our understanding of the thyroid hormone-induced molecular events that lead to time and space-specific conversion of chondrocytes into osteoblasts could lead to potential breakthroughs in terms of identifying novel therapeutic strategies to heal non-union fractures. 2) The default route of chondrocyte differentiation is predicted to be terminal differentiation leading to bone formation.^98,99^ In the articular cartilage, this default route is somehow blocked to obtain permanent articular cartilage. During mechanical injury, inflammation, or aging, the signals that contribute to this blockade are dysregulated leading to a loss of undifferentiated articular chondrocyte progenitors, thus contributing to the pathogenesis of osteoarthritis. Future understanding of the mechanisms involved in temporal and spatial control of chondrocyte to osteoblast differentiation events during endochondral ossification could lead to the development of strategies to manipulate signals that control these events for a therapeutic benefit in the treatment of joint injury and disease. 3) The current anabolic therapies for osteoporosis are based on promotion of osteoblast functions. If the prediction that chondrocytes contribute to an important source of osteoblasts and bone formation processes during both growth and remodeling turn out to be true, then studying the mechanisms that contribute to chondrocyte-to-osteoblast transdifferentiation will provide exciting new strategic approaches to develop anabolic therapies for osteoporosis and other bone wasting diseases.

## Conclusions

While endochondral ossification seems to proceed using the long-known paradigm, it seems that a transdifferentiation mechanism may be working in conjunction with canonical endochondral ossification to promote bone health. Indeed, it is possible that this process may manifest itself using all the different models mentioned in this review, and that each model has a temporo-regional preference. Further, the redundancy observed with transdifferentiation may account for faster tissue regeneration, explosive bone growth, and secondary ossification prior to vascularization of the tissue. As innovative studies become more prevalent in the literature, the contribution of chondrogenic tissue to new bone may provide new avenues for therapeutics for various osteodegenerative diseases and fracture healing methodologies.

## Future directions

The research on transdifferentiation is still in a rudimentary stage and poses a number of unresolved questions which include: (1) Does chondrocyte-to-osteoblast transdifferentiation contribute to the pathogenesis of diseases such as osteoarthritis and heterotopic ossification? (2) Can targeted therapies which promote chondrocyte-to-osteoblast transdifferentiation be developed to promote healing of large skeletal defects and nonunion fractures? (3) Does thyroid hormone regulation of post-natal development in other tissues involve transdifferentiation? (4) Does transdifferentiation have a broader implication in fields such as cancer, cardiovascular diseases, and diabetes? and (5) Can modulation of transcription factors involved in transdifferentiation be used as an effective framework for direct reprograming between cell types?
